# Dynamics of Space Particles and Spacecrafts Passing by the Atmosphere of the Earth

**DOI:** 10.1155/2013/489645

**Published:** 2013-12-12

**Authors:** Vivian Martins Gomes, Antonio Fernando Bertachini de Almeida Prado, Justyna Golebiewska

**Affiliations:** ^1^Grupo de Dinâmica Orbital e Planetologia, Universidade Estadual Paulista, FEG/UNESP, 12516-410 Guaratinguetá, SP, Brazil; ^2^Instituto Nacional de Pesquisas Espaciais (INPE), 12227-010 São José dos Campos, SP, Brazil; ^3^Astronomical Observatory Institute, Faculty of Physics, Adam Mickiewicz University, 60-286 Poznan, Poland

## Abstract

The present research studies the motion of a particle or a spacecraft that comes from an orbit around the Sun, which can be elliptic or hyperbolic, and that makes a passage close enough to the Earth such that it crosses its atmosphere. The idea is to measure the Sun-particle two-body energy before and after this passage in order to verify its variation as a function of the periapsis distance, angle of approach, and velocity at the periapsis of the particle. The full system is formed by the Sun, the Earth, and the particle or the spacecraft. The Sun and the Earth are in circular orbits around their center of mass and the motion is planar for all the bodies involved. The equations of motion consider the restricted circular planar three-body problem with the addition of the atmospheric drag. The initial conditions of the particle or spacecraft (position and velocity) are given at the periapsis of its trajectory around the Earth.

## 1. Introduction

The close approach between two or more celestial bodies is very much studied in the literature. This phenomenon is involved in astronomical problems like the close approaches of three bodies [1–3], encounters of single stars and hard binaries [4], crossing between planets and a rotating star [5], or encounters between a star and a massive black hole [6]. The study of the dynamics of comets during close approaches is also available in the literature [7, 8]. This phenomenon also has many applications in the astronautical field. Several missions used this technique to gain or lose energy, saving fuel in their maneuvers. One of the most famous applications was the Voyager mission, which visited the exterior planets of the Solar System based on the use of close approaches [9, 10]. Other applications of this maneuver are available in the literature, like the use of swing-bys to send a spacecraft to the giant planets [11–14] or even to the Sun [15], the use of Venus in a trip to Mars [16, 17], the studies to make a three-dimensional close approach to Jupiter to change the orbital plane of the spacecraft [18], the use of the passages by the Moon to increase the energy of the spacecraft [19], and the use of multiple passages by the secondary body to find trajectories linking the primaries or the Lagrangian points [20, 21].

A good description of this maneuver is available in [22]. There are many other researches available in the literature considering this problem, several of them optimizing parameters to obtain some desired results, like combining this approach with impulsive maneuvers [23]. The eccentricity of the primaries was also included in some researches, like [24].

The present research studies the effects of the atmosphere of the Earth on the trajectory of a particle or a spacecraft that makes this close approach with the Earth [25, 26]. The mathematical model considers the problem as a circular restricted planar three-body problem [27] plus the atmospheric drag force. Some other papers also consider the behavior of a cloud of particles making the close approach [28, 29].

The equations of motion are numerically integrated starting at the periapsis in both directions of time, until the particle or spacecraft reaches distances far enough from the Earth and the system Sun-particle can be considered a two-body system. At those two points, one for each direction of time, it is possible to compute the two-body energy Sun-particle before and after the passage by the Earth for each particle. If the atmosphere was not present, this type of maneuver would be the standard swing-by described in the references cited previously. Now, with the inclusion of the atmosphere of the Earth, there is a drag force changing the trajectory of the particle or the spacecraft. This drag force is obtained by assuming dependence with the square of the velocity multiplied by the density of the atmosphere, which is calculated based on an exponential relation with the altitude. The objective of this research is to map the energy before and after this passage for different geometries of the approach, velocity, and altitude of the periapsis.

## 2. Definition of the Problem and Mathematical Model

**Figure 1:**
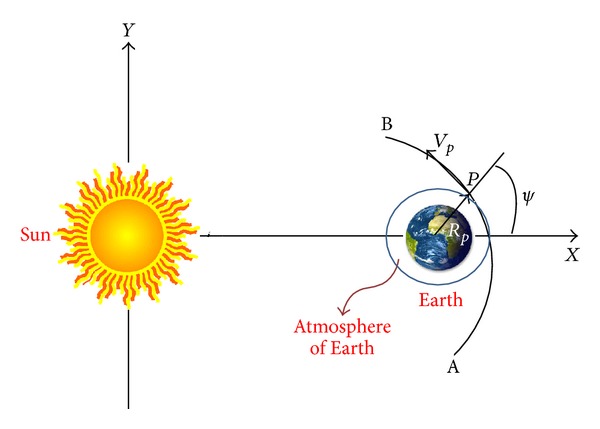
Geometry of the maneuver.

It is assumed that the spacecraft comes from an orbit around the Sun (that can be elliptic or hyperbolic), outside the sphere of influence of the Earth. When approaching the Earth, its trajectory causes the entrance of the particle or spacecraft in the atmosphere and then its motion is governed by the gravity of the planet, assumed to be a point of mass, and the atmospheric drag. The hypothesis for the motion of the Earth is that it is travelling in a circular orbit around the center of mass of the system Sun-Earth. The spacecraft starts its motion in an orbit that is also around the center of mass of the Sun-Earth system.  Figure 1 shows the basic geometry. The particle or spacecraft approaches the Earth from point *A*, makes a close approach with the Earth, including the passage by the atmosphere, and then goes to point *B*. The perigee of the trajectory is marked by point *P*. The atmosphere of the Earth is represented by the circle around its center. At points *A* and *B*, the particle or spacecraft is assumed to be far from the Earth and its motion can be modeled by a two-body problem with the Sun. The equations of motion written in the rotating frame, which is a reference system that rotates together with the Sun-Earth system for each particle or spacecraft, are shown below (see (1) to (4)). The canonical system of units is also used, means that the Sun-Earth distance is the unit of distances, the total mass of the Sun and the Earth is the unit of mass, and the unit of time is defined in such a way that the period of the motion of the Sun-Earth system is 2*π*. So, the equations of motion are [23]

(1) $$\begin{array}{c} \ddot{X}-2\dot{Y}=\frac{\partial \Omega }{\partial X}+F_{X}, \end{array}$$

(2) $$\begin{array}{c} \ddot{Y}+2\dot{X}=\frac{\partial \Omega }{\partial Y}+F_{Y}, \end{array}$$

where *Ω* is the potential, given by

(3) $$\begin{array}{c} \Omega =\frac{1}{2}\left(x^{2}+y^{2}\right)+\frac{\left(1-\mu \right)}{r_{1}}+\frac{\mu }{r_{2}}, \end{array}$$

where *x* and *y* represent the coordinates of the position of the particle/spacecraft; *r*_1_ and *r*_2_ are the distances from the particle/spacecraft to the Sun and the Earth, respectively; *μ* is the gravitational parameter of the Earth; *F*_*x*_ and *F*_*y*_ represent the components of the drag force ($\vec{F}$), which is given by

(4) $$\begin{array}{c} \vec{F}=\left(F_{X},F_{Y}\right)=-C_{B}\rho V\vec{V}, \end{array}$$

where *C*_*B*_ = *C*_*D*_ *A*/2*m* is the ballistic coefficient; *C*_*D*_ is the drag coefficient, which is a parameter that considers the form of the particle or spacecraft; *A* is the cross section area of the particle or the spacecraft; $\vec{V}$ is the velocity of the spacecraft or the particles with respect to the atmosphere; *m* is its mass; *ρ* is the density of the atmosphere, which can be estimated by an exponential function representing a barometric equilibrium:

(5) $$\begin{array}{c} \rho =\rho_{0}e^{-(h-h_{0})/H}. \end{array}$$

In (5), *ρ*_0_ is the density of the atmosphere at an altitude *h*_0_; *h* is the altitude of the spacecraft; *H* is a constant that specifies the decay velocity of the density with the altitude. This is a usual approximation for the density of the atmosphere of the Earth, and the values of the constants involved are obtained by observations.

## 3. Identifying One Trajectory

The set of variables used to define each trajectory is the following:

- *ψ*: the angle of approach, that is, the angle between the line of the periapsis of the trajectory of the spacecraft around the Earth and the line that connects the Sun and the Earth;
- *v*_*p*_: the velocity of the particle or spacecraft at the periapsis;
- *h*_*p*_: the altitude of the periapsis of the trajectory of the particle or spacecraft around the Earth.

So, the sequence of steps shown as follows can show the importance of the drag in the trajectories.

- A numerical integration is performed in backward time, with the particle or spacecraft starting at the periapsis until it reaches point *A* (Figure 1). This point is far enough from the Earth, so the two-body celestial mechanics can be used to obtain the two-body energy Sun-particle or spacecraft before the close approach.
- Then, the numerical integration is made again, with the particle or spacecraft starting at the periapsis again, but this time in forward time. This process is done until the particle or spacecraft collides with the planet or reaches point *B* (see Figure 1), which is assumed to be far from the Earth such that the motion is again Keplerian and the two-body energy Sun-particle or spacecraft after the close approach is obtained.

So, by repeating this strategy for a large range of values of the ballistic coefficient, it is possible to understand the importance of the drag force in the process. The numerical integration is made using a Fourth order Runge-Kutta with step-size control and a numerical precision of 10^−8^.

## 4. Results

**Figure 2:**
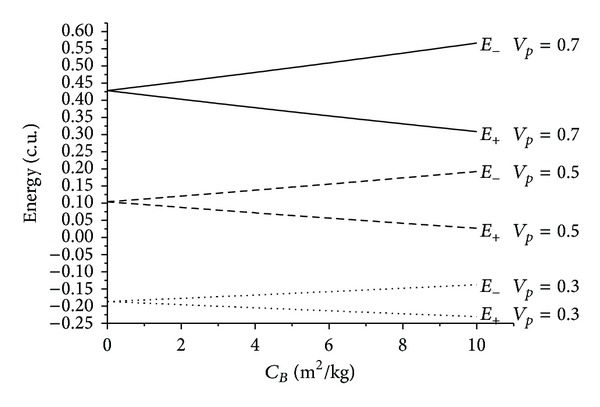
Evolution of the energies as a function of the ballistic coefficients for *ψ* = 0°.

**Figure 3:**
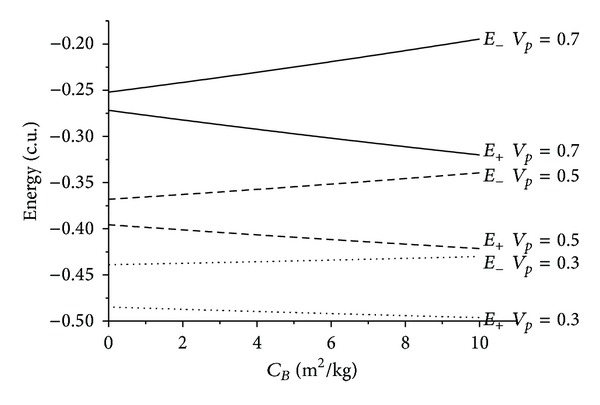
Evolution of the energies as a function of the ballistic coefficients for *ψ* = 90°.

**Figure 4:**
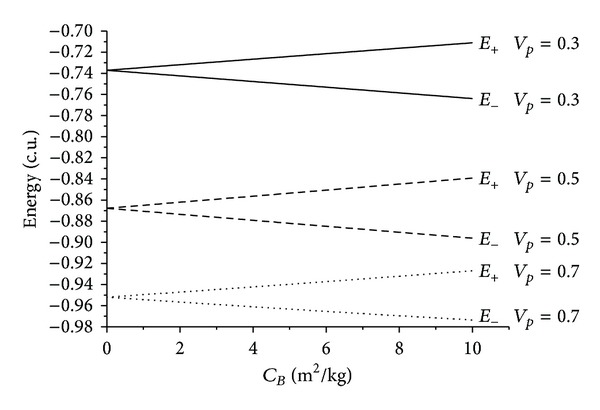
Evolution of the energies as a function of the ballistic coefficients for *ψ* = 180°.

**Figure 5:**
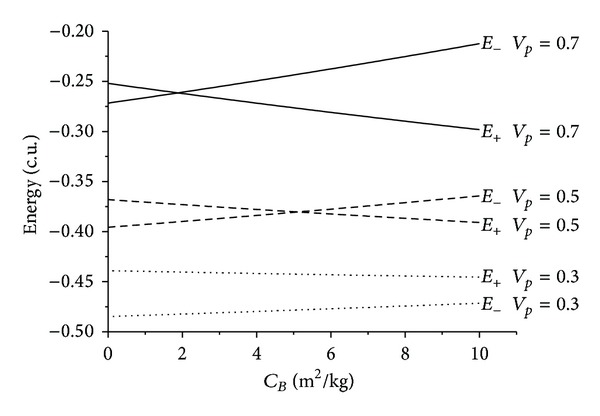
Evolution of the energies as a function of the ballistic coefficients for *ψ* = 270°.

**Figure 6:**
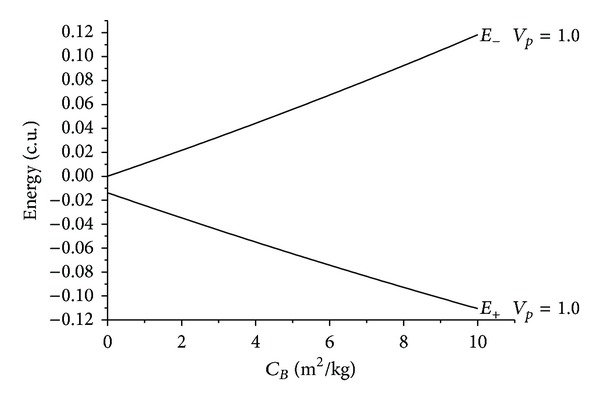
Evolution of the energies as a function of the ballistic coefficients for near-parabolic orbits for *ψ* = 90°.

**Figure 7:**
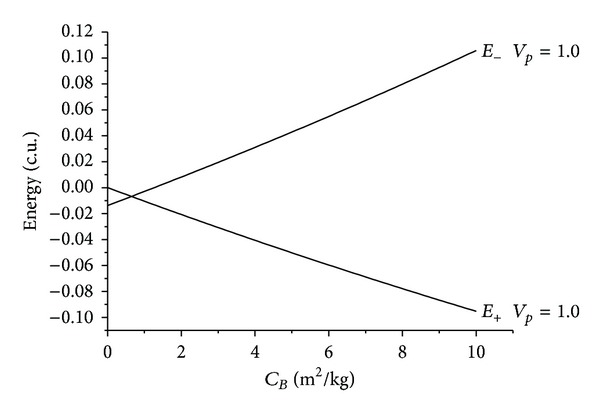
Evolution of the energies as a function of the ballistic coefficients for near-parabolic orbits for *ψ* = 270°.

**Figure 8:**
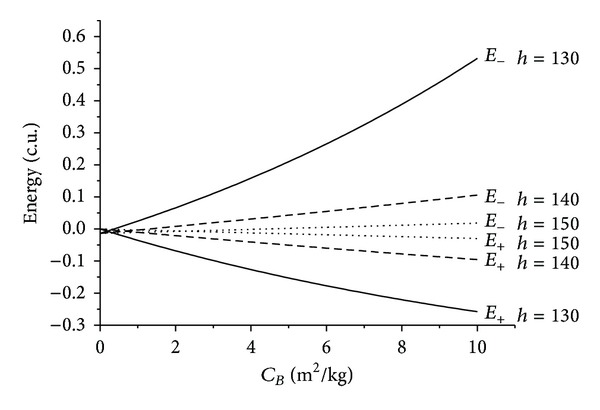
Evolution of the energies as a function of the ballistic coefficients for different values of the periapsis altitude for *ψ* = 270°.

The results are shown in the figures that display the ballistic coefficient (in m^2^/kg) in the horizontal axis and the energy before and after the close approach (in canonical units) in the vertical axis. Several values were considered for the velocity at periapsis *v*_*p*_: 0.3, 0.5, and 0.7 canonical units, where 1.0 canonical unit is equal to 29.78 km/s, that is, the velocity of the Earth in its motion around the Sun. Those values were chosen after making several simulations with different values. It is important to choose values that are not too low, because that causes too many captures by the Earth, and also those which are not too high, which causes the Swing-Bys to have very few effects on the trajectory of the spacecraft. Four values were used for the angle of approach *ψ*: 0°, 90°, 180°, and 270°. The idea was to consider values that cover the particular cases like maximum gains and losses of energy and regions with no effects from the Swing-Bys. Figures 2, 3, 4, 5, 6, 7, and 8 show the results, where *E*_−_ and *E*_+_ represent the energy before and after the Swing-By, respectively. The values for *C*_*B*_ go from zero (a satellite with *C*_*D*_ = 0), that represents a situation where the effects of the drag are not present, to 10 m^2^/kg, that would represent a satellite with a large area/mass ratio, in the order of 1000 m^2^ for a 100 kg satellite, assuming *C*_*D*_ = 2. It implies the use of a panel in the satellite. The value of *C*_*D*_ varies from 2 to 2.3, depending on the interaction of the atmospheric constituents with the satellite surface [30].

There are several facts to be observed from those results. First, it is noticed that the presence of the atmospheric drag increases the variation of energy, always removing energy from the particle in both senses of time. The relation between the *C*_*B*_ and the energy is almost linear for all the situations simulated here. The inclination of those lines increases with the velocity at the periapsis, because the drag force is proportional to the square of the velocity of the particle or spacecraft. This means that a higher value for the velocity at the periapsis causes higher values for the velocities during the whole trajectory inside the atmosphere, so the loss of energy is larger.

Considering, in some detail, the specific cases, it is clear that the maneuvers where *ψ* = 0° and *ψ* = 180° have no change in the energy due to the Swing-By itself. The lines for the energy before and after the passages start at the same points, which means that, for *C*_*B*_ = 0 (situation with no drag) the variation of energy is zero. This is in agreement with the literature [22]. It is also clear that the orbits are all elliptic because the energy is negative before and after the close approach. Another interesting characteristic is that there is an inversion in the order of the lines. For *ψ* = 0° the upper lines represent the higher velocities and for *ψ* = 180° the upper lines represent the lower velocities. This fact can be explained by the geometry of the problem. The energy is related to the two-body system Sun-particle or spacecraft and the velocities are measured with respect to the Earth. So, to obtain the energy, it is necessary to make the vectorial addition of the velocities of the particle or spacecraft with respect to the Earth with the velocity of the Earth with respect to the Sun. The velocity of the Earth with respect to the Sun is always pointing towards the vertical axis, in the positive direction. The velocity of the particle or spacecraft with respect to the Earth points towards the positive direction of the vertical axis when *ψ* = 0°, so both velocities have the same sense and their magnitudes added together cause an increase in the energy with the increase of the velocity at the periapsis. In the situation where *ψ* = 180°, the velocity of the particle with respect to the Earth is in the negative direction of the vertical axis, so the velocities have opposite senses and their magnitudes have to be subtracted from each other to get itsvalue with respect to the Sun, causing a decrease in the energy with the increase of the velocity at the periapsis.

It is also noted that the situation where *ψ* = 0° shows the presence of hyperbolic and elliptic orbits, depending on the velocity at the periapsis.

The maneuvers with *ψ* = 90° represent the conditions that give the maximum loss of energy due to the Swing-by. This loss can be noted by the gap in the initial points of both energies before and after the passage. Note that this gap increases when the velocity at the periapsis decreases, due to the fact that lower velocities allow longer interactions between the bodies, and so the variation of energy is stronger.

On the opposite side, maneuvers with *ψ* = 270° represent the situations that give the maximum gain of energy due to the Swing-by. Once again the gaps in the initial points show that fact. These gaps also increase when the velocity at periapsis increases, for the same reasons already explained. Another characteristic of this geometry is the crossing of the lines of energy before and after the close approach. It happens because the case with *ψ* = 270° and no drag (*C*_*B*_ = 0) has an increase in the energy, so the line of the energy before the maneuver starts at a point higher than the line of the energy after the maneuver. When considering the presence of the atmospheric drag, the loss in the energy starts to occur, and this loss increases with the ballistic coefficient. So, there are values for the *C*_*B*_, marked by the crossing of the lines of the energy before and the energy after the maneuver, where the loss due to the atmospheric drag is equal to the gain due to the Swing-By and the net result is a maneuver with zero variation of energy. This point can even be used for a spacecraft that needs to realize a close approach with the Earth to complete the goals of the mission but does not want to change its orbit. After this point, the loss of energy is larger and the net result is a modification of the orbit that reduces its energy.

After that, a more detailed study is made for near-parabolic orbits. Those are the orbits with two-body energy Sun-particle or spacecraft near zero in the situation without drag. In the situation where *ψ* = 90°, that causes loss of energy due to the Swing-By, the initial orbit is hyperbolic (positive energy) and the final orbit is elliptic (negative energy). This is a situation where a capture occurred. The difference in those energies increases with the ballistic coefficient, so the presence of the atmospheric drag works in favor of those captures and orbits with larger energy can be captured with the help of the atmosphere. At the same time, those orbits become even more closed, with lower values of the energy.

On the opposite side, the situation where *ψ* = 270° generates gains of energy due to the Swing-By. Considering the case with no drag (*C*_*B*_ = 0), the initial orbit is elliptic (negative energy) and the final orbit is hyperbolic (positive energy). This is a situation where an escape occurred. This also is in agreement with the literature [22]. When adding the presence of drag, the mechanism of removal of the energy by the atmosphere starts. Once again there is a point where the variation of energy is zero, as a consequence of the equilibrium between the gain due to the swing-by and the loss due to the atmosphere. The difference among those energies increases with the ballistic coefficient, as expected. So, the presence of the atmosphere causes captures, even in this geometry.

After that, a study is made to take into account the effects of the periapsis altitude in the passage of the particles or the spacecraft by the atmosphere. The simulations are made for the velocity of the periapsis of 1.0 canonical units and angle of approach *ψ* = 270°. Other situations were simulated with similar results, so they are not shown here. The values used for the periapsis altitude are 130, 140, and 150 km above the surface of the Earth.  Figure 8 shows the results. As expected, the effects of the passage are much stronger for lower values of the periapsis altitude because the density of the atmosphere is larger. In that sense, the results quantify these effects and show that the variation in energy is about four times larger when the periapsis is 130 km when compared to the situation where the periapsis is 140 km and fifteen times larger when compared to the value of periapsis altitude of 150 km. Another characteristic shown in the results is that the relation between energy and ballistic coefficient is near linear in most of the cases, but it starts to become quadratic when the periapsis distance decreases. This is a consequence of the exponential model for the density, since the distances are becoming larger and linear approximation of the density is no longer valid.

## 5. Conclusions

A numerical sequence of steps to measure the effects of the atmosphere in a close approach between a particle or a spacecraft with a planet that has an atmosphere is developed and implemented. This approach is then applied to a particle or a spacecraft passing by the atmosphere of the Earth. The results show regions where escapes and captures occur and also quantify the effects of the atmosphere in the trajectories. The atmosphere reduces the energy of the particles or spacecraft, helping captures to occur, even for geometries where the energy should increase due to the close approach. The effects of the velocity at the periapsis are measured in all the situations, in order to quantify its effects by the present algorithm. Near-parabolic orbits are studied in more detail, to emphasize the study of the mechanism of captures of the particles or spacecraft by the atmosphere of the Earth. The study of the variation of the altitude of the periapsis is also made, showing the limits where the relation between energy and ballistic coefficients is linear. On the astronautical side, the present research can be used to help to plan missions with the goal of capturing a spacecraft by the atmosphere of the Earth, giving numerical results that help to define values for the ballistic coefficient to complete a capture. In terms of astronomical problems, the algorithm developed here can help to predict the percentage of captured particles as a function on the initial conditions at the periapsis or before the close approach, by using the values obtained after the backward integration.
